# Evaluation of fractional carbon dioxide laser alone versus its combination with betamethasone valerate in treatment of alopecia areata, a clinical and dermoscopic study

**DOI:** 10.1007/s00403-022-02393-5

**Published:** 2022-09-17

**Authors:** Dalia Abdel Halim, Mariam Nayer, Solwan Ibrahim El-Samanoudy, Heba Mohamed Abdel Raheem, Nanis Ragab

**Affiliations:** Dermatology Department, Faculty of Medicine, Cairo University, Cairo, Cairo 11956 Egypt

**Keywords:** Alopecia areata, Fractional, Carbon dioxide, Laser, Betamethasone valerate

## Abstract

Alopecia areata (AA) is a non-scarring tissue-specific autoimmune disorder. Many therapeutic modalities are available for the treatment of AA, but none has yet proven to be uniformly effective. Fractional carbon dioxide (FRCO_2_) laser has been introduced as a treatment modality for AA. The objective is to evaluate and compare the efficacy and safety of FRCO_2_ laser in treatment of AA alone or in combination with betamethasone valerate cream. 30 patients were assigned to one of the following groups, Group A FRCO_2_, Group B FRCO_2_ plus betamethasone valerate cream or Group C (betamethasone valerate cream). Patients received eight laser sessions 2 weeks apart, treatment period was 4 months. A statistically significant decrease in SALT score, dystrophic hair and a statistically significant increase in terminal hair was observed in all groups. Patient satisfaction level and reduction in SALT score were significantly higher among FRCO_2_ and FRCO_2_ plus betamethasone valerate group. However, no statistical significant difference was found between FRCO_2_ group and FRCO_2_ combined with betamethasone valerate cream group. FRCO_2_ laser is a safe and effective treatment modality for AA when used alone or in combination with betamethasone valerate cream. However, it was found superior to betamethasone valerate cream monotherapy.

## Introduction

Alopecia areata (AA) is a complex genetic immune-mediated inflammatory non-scarring hair loss that results in decrease in the quality of life [11]. Many treatment options including topical, systemic, injectable and laser modalities have been used for the treatment of AA [4]. One of the most unique treatments of alopecia areata is ablative fractional laser treatment with few to no side effects**.** It has been suggested that fractional laser could act by inducing T cell apoptosis, increasing blood flow and promoting telogen to anagen transitions. Minor trauma and wound healing can drive hair growth [13]. A murine model study showed anagen induction related to Wnt/β-catenin pathway after fractional CO_2_ laser treatment, resulting in hair regrowth and increased proportion of anagen hairs. It also found an increase in the mRNA expression of various growth factors associated with wound healing including vascular endothelial growth factor (VEGF), transforming growth factor β 1 (TGF-β1), and keratinocyte growth factor (KGF) and subsequently increased Wnt10-b [1].

The aim of the current study was to evaluate and compare the efficacy of fractional carbon dioxide (FRCO_2_) laser alone versus FRCO_2_ laser in combination with topical betamethasone valerate cream versus topical betamethasone valerate cream monotherapy in the treatment of alopecia areata.

## Patients and methods

In the current pilot study patients with a diagnosis of alopecia areata were recruited from the Dermatology Outpatient Clinic, Kasr al Ainy hospital after approval of dermatology department research ethical committee.

### Patients

A total of 30 patients with alopecia areata of both sexes, aged > 18 years were included in the study. Exclusion criteria were the following, children ≤ 18 years, pregnancy and lactation, alopecia totalis/universalis, patients who received topical or systemic treatment in the 3 last months and history of hypertrophic scars or keloid formation.

### Baseline evaluation

An informed written consent for participation was signed by all patients. Detailed history was taken for all patients including personal, medical and family history as well as previous treatment. Fitzpatrick skin phototype was determined. Scalp was examined and the extent was calculated according to severity of alopecia tool (SALT) score [9]. Dermoscopic examination was done for involved scalp areas to confirm the diagnosis and evaluate severity. Face and body hair were examined to detect involvement. Nails were examined to detect nail dystrophy. Baseline clinical photographs and dermoscopic photos for involved scalp areas.

### Treatment protocol

Patients were assigned to one of the following treatment groups in which all involved patches were treated, Group A: fractional CO_2_ Laser, Group B: fractional CO_2_ Laser plus betamethasone valerate cream and Group C: betamethasone valerate cream only. Treatment period was 4 months.

### Laser treatment and postoperative care

Patients received a total of eight sessions 2 weeks apart using DEKA SmartXide Fractional CO_2_ laser machine. Patients applied local anesthetic cream (Pridocaine cream, which contains lidocaine and prilocaine as active ingredients) 1 h prior to the session. The areas of AA were cleaned with normal saline. The following parameters were used: Power: 16, Dwell time: 600, Spacing: 600, Stack: 2 (Fluence: 2.13 J/cm^2^). After sessions patients were instructed to apply a topical antibiotic (Fusidic acid cream) twice daily for 1 week and apply a broad spectrum sunscreen.

### Topical corticosteroid therapy

Betamethasone valerate cream (Betaval cream) was applied immediately after the laser session and twice daily in between sessions for Group B patients. Group C patients applied cream twice daily.

### Follow-up after treatment

Each patient was assessed clinically and dermoscopically every 2 weeks to ensure compliance, to follow-up treatment response and to detect any side effects. At the end of treatment period photographs were taken, SALT score was done. Physician global assessment was done by two investigators blinded to the used modality, comparing digital photographs before and after treatment using a quartile scale (1: < 25% = no or minimal improvement, 2: > 25–49% = moderate improvement, 3: > 50–74% = marked improvement, 4: > 75–99% = excellent improvement, 5: > 100% = complete improvement). Patient’s overall satisfaction level was determined using a five-point scale (− 1: condition worsened, 0: not satisfied, 1: poorly satisfied, 2: moderate, 3: excellent). Patients were followed-up clinically and dermoscopically 2 months after stoppage of treatment to detect new lesions or hair loss at previously treated areas.

### Trichoscopic assessment

Trichoscopic assessment was carried out at each visit to identify signs of early response. Digital photographs of trichoscopic fields were obtained at baseline and after stoppage of treatment using handheld dermoscope DermLite DL4 attached to a smart phone using a magnetic connection. Photos were evaluated by an independent investigator who was blinded to therapeutic modalities. Two grading scores were used, one to estimate the percentage of empty follicular units/dystrophic hair forms (yellow dots, black dots, broken hairs and exclamation mark hairs) in the representative fields of the patch area (0: no/empty follicular units or dystrophic hair in field, 1: 1–24%, 2: 25–49%, 3: 50–74%, 4: 75–100%). A similar grading score was used to determine the percentage of terminal hairs in the patch area (0: no terminal hair in field, 1: 1–24%, 2: 25–49%, 3: 50–74%, 4: 75–100%). If the patient had multiple patches, each one was evaluated separately, and a mean value was obtained.

### Statistical methods

Data were coded and entered using the statistical package for the Social Sciences (SPSS) version 25 (IBM Corp., Armonk, NY, USA). Comparisons between quantitative variables were done using the non-parametric Kruskal–Wallis and Mann–Whitney tests. For comparison of serial measurements within each patient the non-parametric Wilcoxon signed rank test was used. For comparing categorical data, chi-squared test was performed. Exact test was used instead when the expected frequency is less than 5. Correlations between quantitative variables were done using Spearman correlation coefficient. *p* values less than 0.05 were considered as statistically significant.

## Results

30 patients with alopecia areata were included in the study. They were equally assigned to one of three treatment groups (10 patients each), 27 patients (90%) were males and 3 (10%) were females. Age ranged between 19 and 50 years (mean = 31.23 ± 8.62 years). The duration of disease ranged from 5 to 48 months, mean = 11.47 ± 9.10 months. 11 (36.7%) patients were skin phototype III and 19 (63.3%) were phototype IV Table 1. As regards baseline severity mean SALT was 15.59 ± 12.75. All three groups were strictly matched as regards age, sex, disease duration and history of recurrence.

**Table 1:** Demographic and clinical data of patients

Sex, *n* (%)	
Males	27 (90%)
Females	3 (10%)
Skin phototype, *n* (%)	
III	11 (36.7%)
IV	19 (63.3%)
Age (years)	
Mean ± SD	31.23 ± 8.62
Range	19–50
Duration of disease (months)	
Mean ± SD	11.47 ± 9.10
Range	5–48
Previous episodes, *n* (%)	
Yes	20 (66.7%)
No	10 (33.3%)
Previous treatment *n* %	
Yes	20 (66.7%)
No	10 (33.3)
Precipitating factor (stress) *n*%	
Yes	13 (43.3%)
No	17 (56.7%)
Family history *n*%	
Yes	3 (10%)
No	27 (90%)
Ophiasis *n*%	
Yes	12 (40%)
No	18 (60%)

**Fig. 1:**
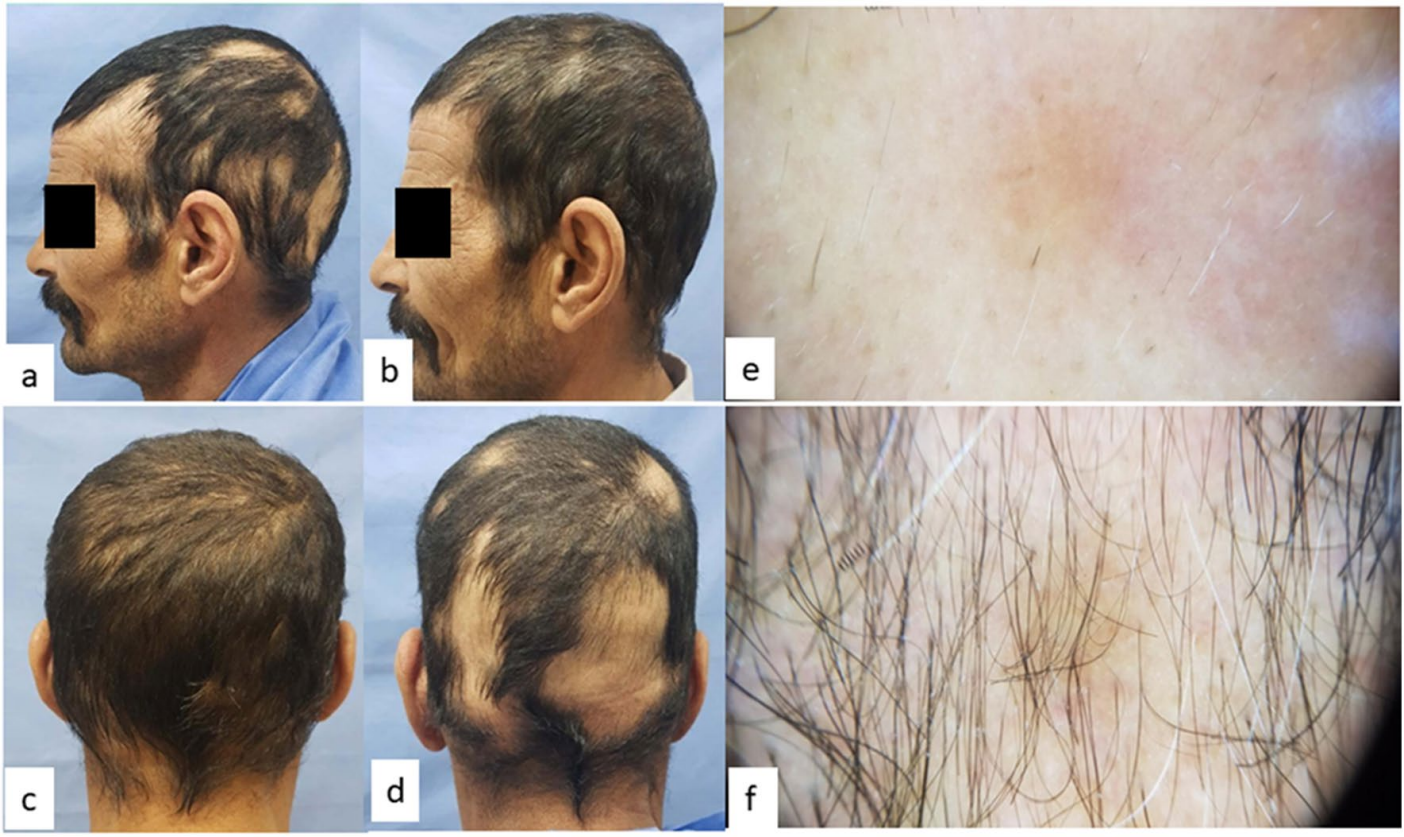
Group A (FrCO_2_): **a** lateral view at baseline, **b** lateral view after treatment, **c** occipital view after treatment, **d** occipital view at baseline, **e** baseline trichoscopy shows dystrophic hair (exclamation hairs and yellow dots grade 4 (75–100%) and no terminal hairs, **f** trichoscopy after treatment shows dystrophic hair grade 2 (25–49%) and terminal hair grade 3

Group A (FRCO_2_) showed a statistically significant decrease in SALT *p* value = 0.005, a statistically significant decrease in dystrophic hair percentage *p* value = 0.004 and a statistically significant increase in terminal hair percentage *p* value = 0.003 (Table 2, Fig. 1). Eight patients started hair regrowth after 3rd session, two patients after 5th session.

**Table 2:** SALT score, dystrophic and terminal hair in Group A (FrCO_2_) before and after treatment

	Mean ± SD	Median	Minimum	Maximum	*p* value
SALT before treatment	22.60 ± 14.93	21	3	44	0.005
SALT after treatment	7.35 ± 8	4.84	0	25	

Dystrophic hair /follicular units	Before treatment count (%)	After treatment count (%)	*p* value
Zero	0 (0%)	3 (30%)	0.004
1–24	0 (0%)	1 (10%)	
25–49	1 (10%)	3 (30%)	
50–74	4 (40%)	3 (30%)	
75–100	5 (50%)	0 (0%)	

Terminal hair	Before treatment count (%)	After treatment count (%)	*p* value
Zero	4 (40%)	0 (0%)	0.003
1–24	4 (40%)	0 (0%)	
25–49	2 (20%)	4 (40%)	
50–74	0 (0%)	1 (10%)	
75–100	0 (0%)	5 (50%)	

*p* value < 0.05 is significant

**Fig. 2:**
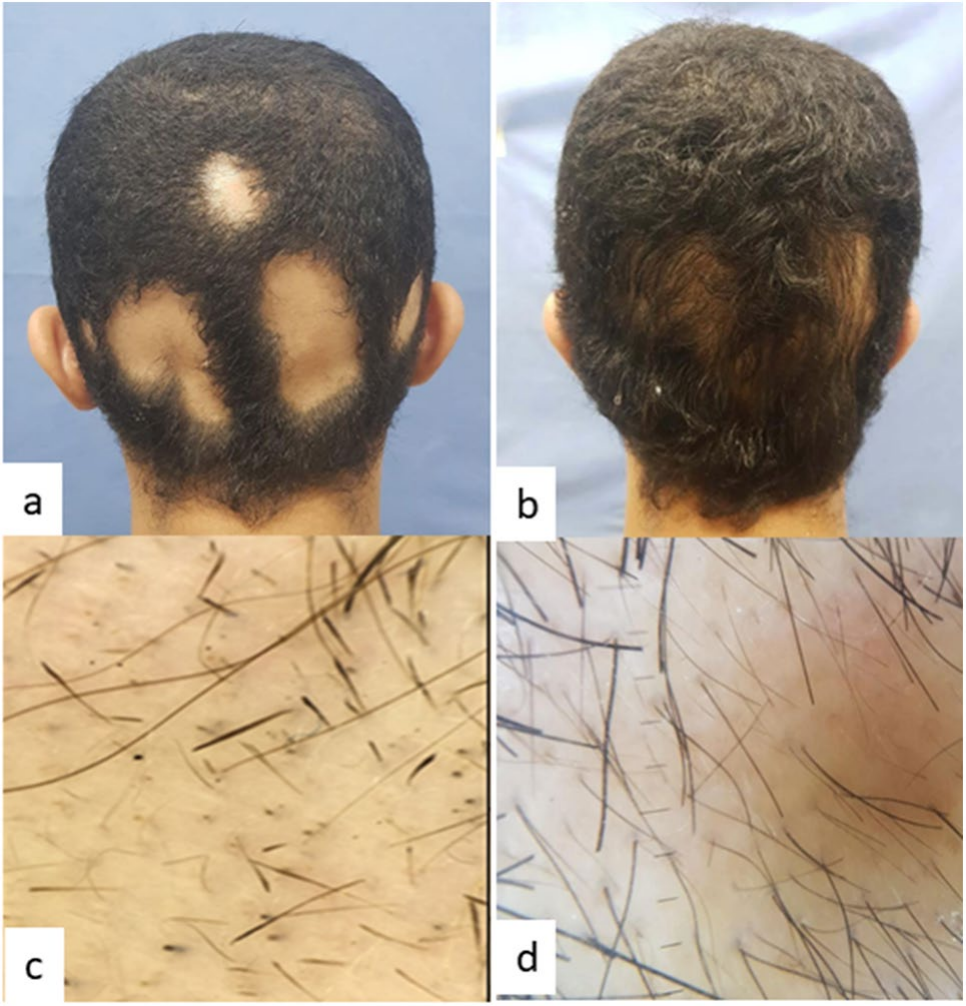
Group B (FrCO_2_ + betamethasone valerate): **a** occipital view at baseline, **b** occipital view after treatment, **c** baseline trichoscopy shows empty follicular openings and dystrophic hair grade 3 (50–74%) and terminal hair grade 1 (1–24), and **d** trichoscopy after treatment shows dystrophic hair grade 2 (25–49%) and terminal hair grade 4 (75–100)

Group B (FRCO_2_ + betamethasone valerate) showed a statistically significant decrease in SALT *p* value = 0.005, a statistically significant decrease in dystrophic hair percentage *p* value = 0.005 and a statistically significant increase in terminal hair percentage *p* value = 0.004 (Table 3, Fig. 2). One patient showed a clinically visible response after 1st session, eight patients after 3rd session and one patient after 4th session.

**Table 3:** SALT score, dystrophic and terminal hair in Group B (FrCO_2_ + betamethasone valerate) before and after treatment

	Mean ± SD	Median	Minimum	Maximum	*p* value
SALT before treatment	18.05 ± 11.10	16.5	5	41.5	0.005
SALT after treatment	6.52 ± 7.95	3.45	0	26	

Dystrophic hair/follicular units	Before treatment count (%)	After treatment count (%)	*p* value
Zero	0 (0%)	3 (30%)	0.005
1–24	0 (0%)	1 (10%)	
25–49	1 (10%)	4 (40%)	
50–74	2 (20%)	2 (20%)	
75–100	7 (70%)	0 (0%)	

Terminal hair	Before treatment count (%)	After treatment count (%)	*p* value
Zero	2 (20%)	0 (0%)	0.004
1–24	7 (70%)	0 (0%)	
25–49	0 (0%)	3 (30%)	
50–74	1 (10%)	3 (30%)	
75–100	0 (0%)	4 (40%)	

*p* value < 0.05 is significant

**Fig. 3:**
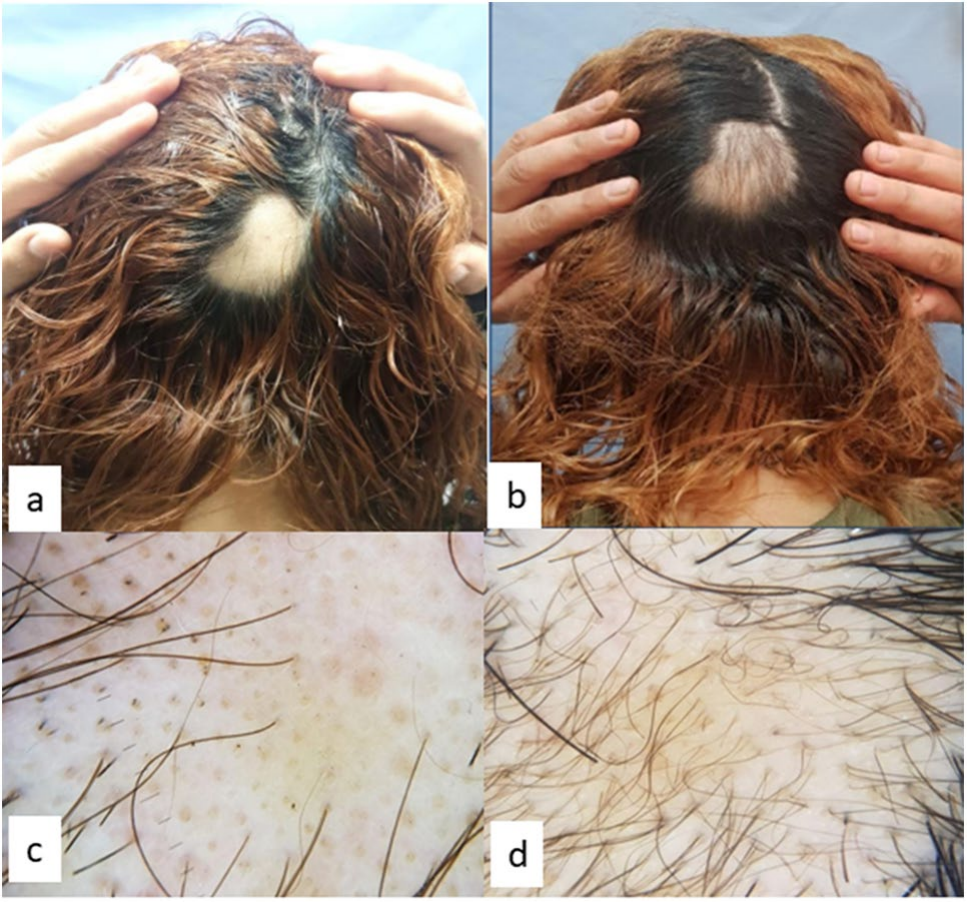
Group C (betamethasone valerate) patient: **a** occipital view at baseline, **b** occipital view after treatment, **c** baseline trichoscopy at shows dystrophic hair (exclamation mark) grade 3 (50–74%) and terminal hair grade 1 (1–24%), and **d** trichoscopy after treatment shows no dystrophic hairs and terminal hair grade 3 (50–74%)

Group C (betamethasone valerate) showed a statistically significant decrease in SALT *p* value = 0.005, a statistically significant decrease in dystrophic hair *p* value = 0.009 and a statistically significant increase in terminal hair *p* value = 0.009 (Table 4, Fig. 3). Six patients showed a clinically visible response after 4 weeks, three patients after 6 weeks and one patient after 8 weeks.

**Table 4:** SALT score, dystrophic hair and terminal hair in Group C (betamethasone valerate) before and after treatment

	Mean ± SD	Median	Minimum	Maximum	*p* value
SALT before treatment	6.13 ± 4.14	6.30	0.24	13.20	0.005
SALT after treatment	3.30 ± 3.64	1.96	0	8.80	

Dystrophic hair/follicular units	Before treatment count (%)	After treatment count (%)	*p* value
Zero	0 (0%)	3 (30%)	0.009
1–24	0 (0%)	0 (0%)	
25–49	0 (0%)	2 (20%)	
50–74	2 (20%)	5 (50%)	
75–100	8 (80%)	0 (0%)	

Terminal hair	Before treatment count (%)	After treatment count (%)	*p* value
Zero	1 (10%)	0 (0%)	0.009
1–24	6 (60%)	1 (10%)	
25–49	3 (30%)	3 (30%)	
50–74	0 (0%)	4 (40%)	
75–100	0 (0%)	2 (20%)	

*p* value < 0.05 is significant

As regards side effects all patients who received FrCO_2_ laser (Groups A and B) reported slight discomfort during procedure and transient post-treatment scaling and erythema, other possible side effects such as secondary bacterial infection and pigmentary changes were not observed. No adverse effects were observed in the topical steroid group (Group C).

Comparing the three groups the reduction in SALT was statistically significant higher among group A (FrCO_2_) compared to C (betamethasone valerate) (*p* = 0.002) and in Group B (FRCO_2_ + betmamethasone valerate) compared to C (*p* = 0.003). However no statistically significant difference was found between Groups A and B (*p* = 1.00). The percentage of reduction in SALT for Groups A, B and C was (67%, 63.81%, 46%), respectively. No statistically significant difference between the three groups was found as regards decrease in dystrophic hair after treatment *p* value = 0.846, increase in terminal hairs after treatment *p* value = 0.389 and physician global assessment *p* value = 0.216. However patient satisfactory level was statistically significant higher in groups A and B compared to C *p* value = 0.039.

No statistically significant correlation was found between duration of disease and SALT, Dystrophic hair percentage, terminal hair percentage and PGA after treatment (*p* = 0.362, *p* = 0.097, *p* = 0.472, *p* = 0.346) respectively.

## Discussion

The current study showed a statistically significant decrease in SALT score, a statistically significant decrease in dystrophic hair and a statistically significant increase in terminal hair with patient satisfaction in FRCO_2_ group. This agrees with a previous report of complete hair regrowth in a 35-year-old male with AA after 6 months of weekly FRCO_2_ sessions [13]. Cho et al., evaluated the clinical effects of combining erbium glass laser and FRCO_2_ laser on three patients with AA, two patients showed marked improvement according to physician global assessment while one patient showed no change [2]. T cell apoptosis, increasing blood flow and promoting telogen to anagen transitions have been proposed as mechanisms by which FRCO_2_ induces hair regrowth in AA [1].

On the other hand a previous study found no increase in the mean hair count according to a digital phototrichogram in AA patches treated with 3–6 sessions of FRCO_2_ compared to control patch. However this study was conducted on long standing and treatment refractory AA [12].

Our study showed a statistically significant decrease in SALT score, a statistically significant decrease in dystrophic hair and a statistically significant increase in terminal hair with patient satisfaction in FRCO_2_ plus betamethasone valerate group. Issa et al., paired a single administration of topical triamcinolone with FRCO_2_ laser in five AA patients. All patients exhibited clinical improvement according to a physician quartile improvement scale [6]. Similarly ten resistant cases of AA received three FRCO_2_ laser sessions followed by topical triamcinolone. Seven patients showed excellent response to the treatment [8].

The generation of microscopic thermal zones (MTZ) by fractional lasers provides channels for a uniform and controlled delivery of drugs. Fractional lasers can penetrate up to 2–3 mm into the dermis, depositing thermal energy where the dermal papilla is, which is where the capillaries surround the hair germ cells [2, 13]. The direct therapeutic effect of fractional laser along with transepidermal drug delivery into the target hair follicles can explain the synergistic effect of fractional laser with topical betamethasone valerate used in this study. An additional benefit is the avoidance of pain and side-effects associated with repeated multiple intradermal injections of corticosteroid.

The current study showed a statistically significant decrease in SALT score, a statistically significant decrease in dystrophic hair and a statistically significant increase in terminal hair with patient satisfaction in betamethasone valerate group.

El-Ashmawy et al., found topical betamethasone valerate solution, latanoprost and minoxidil 5% safe and effective in treatment of AA [3]. Although Kuldeep et al., reported superior results with intralesional triamcinolone compared to betamethasone valerate foam and Tacrolimus ointment, betamethasone valerate was found superior to tacrolimus, side effects in the form of pain and skin atrophy were noted in triamcinolone group [7]. Topical corticosteroids act as anti-inflammatory agents decreasing the dense peribulbar infiltrate allowing the hair follicle to restore the normal hair growth cycle [5]. Due to its easy and painless application and wide safety margin, it is considered first line of treatment in children [10].

Comparing the three groups, the reduction in SALT score was statistically significant higher in FRCO_2_ group and FRCO_2_ plus betamethasone valerate compared to betamethasone valerate group. However, no statistically significant difference was found between FRCO_2_ and FRCO_2_ plus betamethasone valerate groups, indicating the efficacy of FRCO_2_ laser as a sole treatment for AA. The addition of betamethasone valerate cream to FRCO_2_ might have reduced the inflammatory response essential for hair regrowth in AA. Patient satisfactory index was statistically significant higher in FRCO_2_ group and FRCO_2_ plus betamethasone valerate group compared to betamethasone valerate group and hair regrowth was faster among group FRCO_2_ group and FRCO_2_ plus betamethasone valerate group. Although physician global assessment (PGA) was found statistically insignificant between the three groups, four patients in the betamethasone valerate group showed minimal or no improvement according to PGA while in the other groups improvement was moderate to complete. In the current study, laser treatment was well tolerated by patients, adverse effects were limited to pain, transient post-treatment erythema, edema and pruritus which agrees with previous studies [8, 13]. None of the patients showed recurrence of alopecia areata at the treated scalp area 2 months after the endpoint.

In the present study, no significant correlation was found between the duration of the disease and different parameters of improvement (decrease in SALT score, decrease in dystrophic hair, increase in terminal hair and physician global assessment) indicating that FRCO_2_ could be used for early or resistant cases of AA [8]. Limitations to the current study are the small sample size, the short follow-up period and the lack of a control patch.

## Conclusion

FRCO_2_ laser is a safe and effective treatment modality for localized AA when used alone or in combination with betamethasone valerate cream. It was found superior to betamethasone valerate monotherapy. For future studies we recommend a larger sample size, a longer follow-up period to evaluate long term treatment outcomes.
